# Gothenburg Empowerment Scale (GES): psychometric properties and measurement invariance in adults with congenital heart disease from Belgium, Norway and South Korea

**DOI:** 10.1186/s12955-022-02056-z

**Published:** 2022-10-20

**Authors:** Mariela Acuña Mora, Koen Raymaekers, Liesbet Van Bulck, Eva Goossens, Koen Luyckx, Adrienne H. Kovacs, Brith Andresen, Ju Ryoung Moon, Alexander Van De Bruaene, Jessica Rassart, Philip Moons

**Affiliations:** 1 Faculty of Caring Science, Work Life and Social Welfare, University of Borås, Borås, Sweden; 2 Institute of Health and Care Sciences, University of Gothenburg, Gothenburg, Sweden; 3 School Psychology and Development in Context, Leuven, KU Belgium; 4 Research Foundation Flanders (FWO), Brussels, Belgium; 5 Department of Public Health and Primary Care, KU Leuven, Kapucijnenvoer 35, B-3000 Leuven, Box 7001, Belgium; 6 Faculty of Medicine and Health Sciences, Centre for Research and Innovation in Care, Division of Nursing and Midwifery, University of Antwerp, Antwerp, Belgium; 7 UNIBS, University of the Free State, Bloemfontein, South Africa; 8 Knight Cardiovascular Institute, Oregon Health & Science University, Portland, OR USA; 9 Department of Cardiothoracic Surgery, Department of Cardiology, Oslo University Hospital, Oslo, Norway; 10 Department of Nursing, Samsung Medical Center, Seoul, South Korea; 11 Congenital and Structural Cardiology, University Hospitals Leuven, Leuven, Belgium; 12 Department of Cardiovascular Sciences, KU Leuven, Leuven, Belgium; 13 Department of Paediatrics and Child Health, University of Cape Town, Cape Town, South Africa

**Keywords:** Adults, Congenital heart defects, Chronic conditions, Measurement invariance, Patient empowerment, Psychometrics, Validity, Reliability

## Abstract

**Background:**

Patient empowerment is associated with improvements in different patient-reported and clinical outcomes. However, despite being widely researched, high quality and theoretically substantiated disease-generic measures of patient empowerment are lacking. The few good instruments that are available have not reported important psychometric properties, including measurement invariance. The aim of this study was to assess the psychometric properties of the 15-item Gothenburg Empowerment Scale (GES), with a particular focus on measurement invariance of the GES across individuals from three countries.

**Methods:**

Adults with congenital heart disease from Belgium, Norway and South Korea completed the GES and other patient-reported outcomes as part of an international, cross-sectional, descriptive study called APPROACH-IS II. The scale’s content (missing data) and factorial validity (confirmatory factor analyses), measurement invariance (multi-group confirmatory factor analyses), responsiveness (floor and ceiling effects) and reliability (internal consistency) were assessed.

**Results:**

Content validity, responsiveness and reliability were confirmed. Nonetheless, metric but not scalar measurement invariance was supported when including the three countries, possibly because the scale performed differently in the sample from South Korea. A second set of analyses supported partial scalar invariance for a sample that was limited to Norway and Belgium.

**Conclusion:**

Our study offers preliminary evidence that GES is a valid and reliable measure of patient empowerment in adults with congenital heart disease. However, cross-country comparisons must be made with caution, given the scale did not perform equivalently across the three countries.

**Registration** ClinicalTrials.gov: NCT04902768.

## Background

Patient empowerment is a concept associated with increasing peoples’ ability to manage their condition and their lives [1]. It initially emerged in the health promotion field and later demonstrated relevance in the care of persons with chronic conditions (CCs) [2]. It has been suggested that by increasing patient empowerment, patients may eventually become more actively engaged in their care, develop more disease-related knowledge, and improve their quality of life and well-being [3].

There is a considerable amount of research on patient empowerment, both theoretically and empirically [4]. However, to date, there is no consensus on how to define patient empowerment or which instrument is best at capturing this construct. There are approximately 30 different definitions available and more than 40 instruments [4]. Moreover, a detailed assessment of current instruments has indicated that a vast majority lack a clear theoretical ground and have poor validity and reliability [5, 6].

Given the methodological limitations of existing instruments, a new measure, the Gothenburg Young Persons Empowerment Scale (GYPES), was developed [7]. Scale construction reflected the theoretical work of Small and colleagues who defined patient empowerment as “an enabling process or outcome arising from communication with the healthcare professional and a mutual sharing of resources over information relating to illness, which enhances the patients’ feelings of control, self-efficacy, coping abilities and ability to achieve change over their condition” [8]. From semi-structured interviews with adults with CCs, they concluded that patient empowerment comprises five dimensions [8]. First, *knowledge and understanding*, related to the level of knowledge patients need to manage their illness and lives. Second, *personal control*, given that each patient should have the ability to manage their disease. Third, *identity*, which entails how much the illness influences the patients’ lives and their sense of self. Fourth, *shared decision-making*, the ability and possibility to make decisions together with the healthcare provider. Fifth, *enabling others*, referring to the ability to share experiences and coping strategies with other persons who are experiencing a similar situation.

GYPES was developed for use with adolescents with CCs because at the time there were no existing instruments developed specifically for this age group. The scale’s content and factorial validity, internal consistency, and responsiveness were evaluated in two samples of young persons with congenital heart disease (CHD) and type I diabetes. GYPES was found to have good psychometric properties in these groups [7].

Given that the GYPES was found to be valid and reliable for its use with adolescents with CCs, and because it was based on sound conceptual grounds, we developed a slightly modified version to measure empowerment in adults with CCs: the Gothenburg Empowerment Scale (GES). In the evaluation of the scale’s psychometric properties reported herein, we also chose to investigate measurement invariance, given potential use of the GES in international studies. Measurement invariance, also known as measurement equivalence, is often neglected in psychometric evaluation [9], yet it is important to investigate whether an instrument measures the same construct in different contexts. If invariance is not supported, this suggests that the instrument triggers different response mechanisms in different groups, making score comparisons invalid [10]. Invariance is particularly important if cross-cultural comparisons are to be made [11].

Therefore, the aim of this study was to assess the psychometric properties of the GES, including the level of measurement invariance among adults with CHD from different cultures.

## Methods

### Development of GES

Development of the GES was modeled after the 15-item GYPES, which includes three items each to assess the five dimensions of patient empowerment: (1) personal control; (2) knowledge and understanding; (3) identity; (4) shared decision-making; and (5) enabling others [8]. Scale items are purposefully disease-generic, to facilitate use within different populations of CCs. Originally created in English, the GYPES has currently been translated to other languages including Swedish, Dutch, Mandarin, and Turkish. Additional information on the development and evaluation of GYPES has been published [7].

The GES includes the same five dimensions and number of items as GYPES. The only modification was changing the term “young persons” by “persons” throughout the scale. To translate the GES to Dutch, Norwegian and Korean for the present study, the translation process followed the guidelines from the World Health Organization [12]. This process entailed a forward-backward translation, pre-testing the translated questionnaires in a few patients, proofreading the questionnaire and finalizing the translation. To assure consistency, substantial changes from the English version were not permitted.

### Design

This methodological study is part of a larger international, cross-sectional descriptive study known as APPROACH-IS II, which aims to increase the understanding of patient-reported outcomes in adults with CHD, by enrolling adults with CHD from 32 different countries across the world [13]. APPROACH-IS II is registered at ClinicalTrials.gov: NCT04902768.

### Sample

APPROACH-IS II is enrolling participants from over 50 adult CHD centers worldwide. Data collection is scheduled to be finished by the end of August 2022. For the present study, data from participants of Belgium, Norway and South Korea were included, since the data collection of these three countries was undertaken before the covid-19 pandemic.

Participants were eligible for the study if they fulfilled the following criteria: (i) diagnosis of CHD; (ii) aged 18 years or older at the moment of inclusion; (iii) diagnosed before the age of 10 years, (because we wanted participants to have experience living with CHD); (iv) in follow-up at an adult CHD center or included in a national/regional registry; and (v) having the physical, cognitive and language abilities to complete the self-report questionnaires. Patients with prior heart transplantation were ineligible.

In the present study, there are a total of 850 people enrolled, 497 were from Belgium, 144 from Norway, and 209 from South Korea. The median age for the total sample was 30 years, and the proportion of men and women was fairly equal. Moderate CHD was found in 56.4% of the participants. Demographic and clinical information of the included participants is detailed in Table 1. There were significant differences in age (p < 0.001, eta square: 0.044) and complexity (p < 0.001, Cramer’s V: 0.227) between the samples, with moderate effect sizes. Indeed, the Norwegian sample was slightly older, and the proportion of severe CHD was larger in South Korea.

**Table 1:** Sociodemographic and clinical aspects

	Total Sample (n = 850)	Belgium (n = 497)	Norway (n = 144)	South Korea (n = 209)
**Age at inclusion (median, (Q1;Q3))**	30.0 (27;43)	29.0 (27;38)	40.0 (30;53)	31.0 (24;45)
**Sex (n (%))** Female Male	430 (51.0) 413 (49.0)	240 (48.3) 253 (50.9)	86 (60.1) 57 (39.9)	104(50.2) 103 (49.8)
**Education (n (%))** No high school education High school Bachelor’s degree Master’s degree or higher	447 (5.2) 346 (41.2) 258 (30.8) 190 (22.6)	29 (5.9) 194 (39.6) 160 (32.7) 106 (21.6)	10 (6.9) 60 (41.7) 33 (22.9) 41 (28.5)	5 (2.4) 92 (44.9) 65 (31.7) 43 (21.0)
**CHD complexity (n (%))** Mild Moderate Severe	116 (13.8) 473 (56.4) 249 (29.7)	98 (19.7) 313 (63.0) 86 (17.3)	8 (6.0) 82 (61.7) 43 (32.3)	10 (4.8) 78 (37.5) 120 (57.7)

### Procedure

Participants completed a set of self-reported questionnaires, including the GES, that were administered during outpatient clinic visits (in Norway, South Korea and Belgium) and/or mailed to their home (in Belgium). Data for this sub-study were collected between August 2019 and February 2020, hence before the COVID-19 pandemic emerged.

### Statistical analyses

The psychometric evaluation of GES entailed an assessment of the content validity, factorial validity, measurement invariance, internal consistency and responsiveness. *Content validity* was assessed by the proportion of missing values and invalid scores [7]. While this is not a common approach for evaluating content validity, missing values can be considered an indicator of whether the items in the scale are perceived as relevant by the participants and whether the items are intelligible. Less than 5% missing data was deemed acceptable in this study.

*Factorial validity* was evaluated through confirmatory factor analyses (CFA) to assess the hypothesized five-factor structure of the scale and the overall factor of patient empowerment [14]. CFA was performed for the entire sample. A good model fit was obtained if the comparative fit index (CFI) was > 0.90, standardized root mean square residual (SRMR) < 0.08 and root mean square error of approximation (RMSEA) < 0.08 [15]. These fit indices were chosen based on Kline’s suggestion for fit evaluation of CFA [14]. Standardized factor loadings are also reported.

*Measurement invariance* was examined through multigroup CFA (MGCFA) across countries [11]. The MGCFA included three models: (1) configural model (i.e., separates the sample into three subgroups, but no parameter constraints are imposed); (2) metric model (i.e., constrains the factor loadings to be equal across subgroups); and (3) scalar model (i.e., constrains the factor loadings and the item intercepts to be equal across subgroups) [16]. These models are tested sequentially and the process begins with a configural model that is well-fitting. Measurement invariance is based on how well the model fits the data as indicated by the fit indices mentioned before (i.e., CFI, RMSEA, SRMR). Additionally, change fit statistics are used to determine whether measurement invariance is present or not. This change refers to how the fit indices increase or decrease as more constraints are added. As per current recommendations, decreases below 0.01 in CFI (∆CFI) and increases below 0.015 for RMSEA (∆RMSEA) and below 0.03 for SRMR (∆SRMR) are deemed acceptable [17]. If measurement invariance was not achieved at some point, modification indices were assessed to determine whether model fit could be improved or partial invariance could be established [18]. Partial invariance occurs when some items that are different across groups are estimated freely, while keeping at least two indicators per latent construct to be equal across groups [9, 10, 19]. For the GES, this meant that only one item per dimension could be freed to attempt to establish partial invariance. If items were released, this was done following a backward method, based on the items with the highest modification indices [10]. Lastly, if no configural, metric or scalar invariance could be achieved, the factor structure was assessed separately in each group [18].

*Internal consistency* was assessed by calculating the Cronbach’s alpha coefficient [20]. Coefficients were calculated for each dimension and for the overall scale. Besides Cronbach’s alpha coefficients, composite reliability values were calculated as an additional method to evaluate the inter-item consistency of the scale. The Cronbach’s alpha value as well as the composite reliability values should be above 0.70 to be considered acceptable [21]. Floor and ceiling effects were calculated as a way to assess issues regarding *responsiveness*. This is an indirect way to evaluate a scale’s sensitivity to detect change [22]. Floor and ceiling effects were considered present if more than 15% of the participants achieved the lowest (i.e. 15) or highest score (i.e. 75) [22].

Statistical analyses were performed with the Lavaan package in R [23] and IBM SPSS Statistics for Windows version 27.

### Ethics and informed consent

APPROACH-IS II has its coordinating center at KU Leuven, Belgium. Therefore, ethical approval was granted from the Institutional Review Board of the University Hospitals Leuven/KU Leuven. Additionally, ethics approval was granted by the local ethics committees of the included centers (i.e., Norway and South Korea). All participants included in this study provided verbal and written informed consent.

## Results

### Content validity

The proportion of missing values ranged from 0 to 0.8% (Table 2). “K*nowledge and understanding*” was the dimension with the highest proportion of missing values.

### Factorial validity

The five-factor structure as well as the overall factor of patient empowerment of the GES were evaluated through CFA in the entire sample. The five-factor model had an acceptable model fit based on the fit indices (*x*^*2*^*(80) =* 326.296; CFI = 0.948; RMSEA = 0.060; and SRMR = 0.039). Factor loadings for this model ranged from 0.515 to 0.864 and were all significant with p < 0.001 (Table 2). Item 6 from the “*personal control*” dimension had the lowest factor loading (i.e., 0.515). A second-order factor model to test the overall construct of patient empowerment also showed an acceptable model fit ( *x*^*2*^*(85)* = 358.916; CFI = 0.942; RMSEA = 0.062; and SRMR = 0.044). The first-order factor loadings in this model ranged from 0.523 to 0.863 and all were significant (p < 0.001). The second-order factor loadings had the following values: 0.903 (knowledge and understanding), 0.859 (personal control), 0.638 (identity), 0.773 (shared decision-making) and 0.583 (enabling others).

**Table 2:** Proportion of missing values and factor loadings for GES

Items	Missing values n^*^ (%)	Factor loadings	
		**First-order factor**	**Second-order factor**
*Knowledge and Understanding*			
1. I know and understand *my medical condition*	7 (0.8)	0.718	0.723
2. I know what to do to stay healthy	3 (0.4)	0.775	0.765
3. I know when to contact healthcare providers for *my medical condition*	4 (0.5)	0.632	0.639
*Personal Control*			
4. I have the skills to manage *my medical condition* in daily life	3 (0.4)	0.779	0.773
5. I have a sense of control over my health	6 (0.7)	0.682	0.683
6. I am active in maintaining my health	0 (0)	0.515	0.523
*Identity*			
7. *My medical condition* is a part of who I am as a person	1 (0.1)	0.692	0.701
8. Living with *my medical condition* makes me stronger as a person	3 (0.4)	0.610	0.595
9. I have given *my medical condition* a place in my life	5 (0.6)	0.642	0.646
*Shared decision-making*			
10. I am capable of expressing to my healthcare providers what is important to me	1 (0.1)	0.715	0.713
11. I actively participate in discussions with my health care providers about my health	4 (0.5)	0.720	0.719
12. I am capable of making decisions about my health and health care with the healthcare providers	2 (0.2)	0.760	0.762
*Enabling Others*			
13. I have the skills to support other people with a similar *medical condition*	3 (0.4)	0.864	0.863
14. I am able to give helpful advice to people who are struggling with a similar *medical condition*	5 (0.6)	0.853	0.853
15. I can help other people by sharing how I keep myself well	1 (0.1)	0.756	0.757

### Measurement invariance

The configural model had a CFI (0.899) and RMSEA (0.085) near the cut-off values for an acceptable model (Table 3). To improve model fit, modification indices were evaluated, and based on this, the residuals of items 6 and 15 were allowed to covary. Even though these items belong to different dimensions (personal control and enabling others, respectively), it is reasonable to expect that persons who are actively involved in their care, also feel more capable of sharing their experiences with others [7]. A second configural model was evaluated with this error correlation, and model fit indices reached the expected threshold (*x*^*2*^*(252) =* 695.911; CFI = 0.913; RMSEA = 0.079; and SRMR = 0.061). By achieving a well-fitting configural model, we proceeded to test metric and scalar invariance, also including this covariation.

The metric model fitted the data well (Table 3). Additionally, changes in model fit indices (∆CFI 0.005; ∆RMSEA 0.002; ∆SRMR 0.006) were within the expected values, indicating metric invariance was supported. An evaluation of scalar invariance came along with slightly worse model fit indices (Table 3). In comparison to the metric model, fit indices were not within the allowed change range (∆CFI 0.034; ∆RMSEA 0.010; ∆SRMR 0.007). Therefore, full scalar invariance could not be established.

Given the lack of full scalar invariance, we proceeded to test partial scalar invariance by evaluating modification indices and the equality constraints across groups. Constraints were relaxed sequentially until an acceptable fit was achieved. Fixing the intercept of item 9 (identity) contributed the most to the observed misfit across groups, so this item was estimated freely in a partial scalar invariance model. While model fit improvements were obtained, the indices were below the acceptable threshold. Therefore, a new partial scalar invariance model was tested with the intercepts of items 9 (identity) and 4 (personal control) set free. While improvements were identified (Table 3), model fit indices were still not within an acceptable range. We continued to release items sequentially to achieve acceptable model fit. Models were tested with intercepts of items 9 (identity), 4 (personal control), 12 (shared decision-making) and 2 (knowledge and understanding) unconstrained and while fit indices were almost within the recommended range, it was not possible to achieve acceptable model fit. While models with unconstrained items from the “enabling others” dimension were assessed, none of them led to changes in the model fit indices. Additionally, given that not more than one item per dimension could be estimated freely, models with more than 5 free items where not tested. Therefore, partial scalar invariance was rejected.

Since partial scalar invariance was not achieved, an assessment of the three groups independently through CFA was undertaken to understand why this was the case. While fit indices for Norway (*x*^*2*^*(85) =* 117.694; CFI = 0.964; RMSEA = 0.053; and SRMR = 0.074) and Belgium ( *x*^*2*^*(85) =* 348.280; CFI = 0.916; RMSEA = 0.079; and SRMR = 0.053) indicated the model fitted these groups well, it appears this was not the case for the data from South Korea (*x*^*2*^*(85)*: 307.753; CFI: 0.788; RMSEA: 0.112; and SRMR: 0.079). Factor loadings for this country ranged between 0.407 and 0.772, with low factor loadings in the “*shared decision-making*” dimension, though significant (p < 0.001).

#### Measurement invariance: norwegian and belgian samples

As the CFA models for Norway and Belgium had an acceptable model fit. An evaluation of measurement invariance within these two countries was undertaken. Configural invariance was supported by a well-fitted model (*x*^*2*^*(170) =* 463.510; CFI = 0.928; RMSEA = 0.074; and SRMR = 0.057). An evaluation of metric invariance also showed acceptable model fit indices (Table 3) and changes in them were also within the acceptable ranges (∆CFI 0.001; ∆RMSEA 0.002; ∆SRMR 0.004). Given that metric invariance was supported, scalar invariance was evaluated next. Fit indexes for this model were within the recommended values (Table 3). However, changes in the CFI were outside of the acceptable range to support measurement invariance (∆CFI 0.013; ∆RMSEA 0.004; ∆SRMR 0.002). Therefore, full scalar invariance was not supported. Modification indices indicated that freeing the intercept of item 11 (shared decision-making) could lead to model improvements. Hence, partial scalar invariance was evaluated with the intercept of item 11 free. This model had acceptable model fit indexes (*x*^*2*^*(192) =* 519.18; CFI = 0.920; RMSEA = 0.073; and SRMR = 0.063). Additionally, in comparison to the metric model, fit indices suggested worse model fit, but changes were still within acceptable ranges (∆CFI 0.007; ∆RMSEA 0.002; ∆SRMR 0.001). These results support partial scalar invariance for the samples of Norway and Belgium.

**Table 3:** Model fit indexes of multi-group confirmatory factor analyses

Model	X^2^ (*df*)	CFI	RMSEA	SRMR	∆X^2^ (∆*df*)	∆CFI	∆RMSEA	∆SRMR
***Complete sample (Belgium, Norway and South Korea)***								
*Configural invariance* ^*a*^	772.074 (255)	0.899	0.085	0.063				
*Configural invariance* ^*b*^	695.911 (252)	0.913	0.079	0.061				
*Metric invariance* ^*b*^	750.492 (280)	0.908	0.077	0.067	54.581 (28)	0.005	0.002	0.006
*Scalar invariance* ^*b*^	941.720 (298)	0.874	0.087	0.074	191.228 (18)	0.034	0.010	0.007
*Partial scalar invariance with intercept of item 9 free* ^*b*^	886.943 (296)	0.885	0.084	0.072				
*Partial scalar invariance with intercept of items 9 and 4 free* ^*b*^	856.568 (294)	0.890	0.082	0.070				
*Partial scalar invariance with intercept of items 9, 4 and 12 free* ^*b*^	827.343 (292)	0.896	0.080	0.069				
*Partial scalar invariance with intercept of items 9, 4, 12 and 2 free* ^*b*^	813.205 (290)	0.898	0.080	0.069				
***Partial sample (Belgium and Norway)***								
*Configural invariance* ^*a*^	463.510 (170)	0.928	0.074	0.057				
*Metric invariance* ^*a*^	482.543 (184)	0.927	0.071	0.061	19.032 (14)	0.001	0.002	0.004
*Scalar invariance* ^*a*^	544.309 (193)	0.914	0.075	0.064	61.767 (9)	0.013	0.004	0.002
*Partial scalar invariance with one intercept free* ^*c*^	519.177 (192)	0.920	0.073	0.063	36.635 (8)	0.007	0.002	0.001

^a^ Model without error correlation; ^b^ Model with error correlation between items 6 and 15. Models were considered to have acceptable fit if CFI > 0.90, RMSEA and SRMR values < 0.08. MGCFA needed also to have it indexes changes within these ranges: ΔCFI < 0.010, ΔRMSEA < 0.015, and ΔSRMR < 0.030

### Internal consistency

The Cronbach’s alpha for the overall scale was 0.873, indicating that it was internally consistent. The alpha values for the subscales were: 0.73 (knowledge and understanding); 0.693 (personal control); 0.680 (identity); 0. 774 (shared decision-making); and 0.863 (enabling others). The Cronbach’s alpha for each country is given in Table 4. An evaluation of the scale’s composite reliability showed that most of the values are above the expected range of 0.7 and those below such threshold were relatively near to this acceptable value.

### Responsiveness

The mean patient empowerment score for the entire sample was 59.36 ± 8.47. None of the participants had the lowest score (i.e., 15) and only 2.5% had the highest attainable score of 75. Hence, no floor or ceiling effects were identified.

**Table 4:** Cronbach’s alpha values, composite reliability and mean empowerment scores

	Total Sample	Belgium	Norway	South Korea
***Cronbach’s alpha values***				
*Knowledge and understanding*	0.730	0.721	0.759	0.728
*Personal control*	0.693	0.633	0.636	0.751
*Identity*	0.680	0.682	0.751	0.636
*Shared decision-making*	0.774	0.833	0.804	0.491
*Enabling others*	0.863	0.872	0.905	0.782
*Overall scale*	0.873	0.878	0.871	0.854
***Composite reliability***				
*Knowledge and understanding*	0.753	0.752	0.779	0.732
*Personal control*	0.702	0.650	0.677	0.753
*Identity*	0.684	0.682	0.770	0.657
*Shared decision-making*	0.559	0.835	0.805	0.470
*Enabling others*	0.864	0.876	0.910	0.782
*Overall scale*	0.929	0.942	0.951	0.917
***Mean patient empowerment scores***				
*Knowledge and understanding*	12.94 (1.90)	13.07 (1.94)	12.93 (2.03)	12.61 (1.68)
*Personal control*	12.04 (2.10)	12.36 (1.97)	12.59 (1.85)	10.90 (2.12)
*Identity*	11.66 (2.39)	11.82 (2.42)	11.19 (2.79)	11.58 (1.97)
*Shared decision-making*	12.15 (2.26)	12.55 (2.22)	11.58 (2.75)	11.61 (1.74)
*Enabling others*	10.57 (2.83)	10.72 (2.90)	10.51 (3.12)	10.25 (2.40)
*Overall scale*	59.37 (8.47)	60.52 (8.53)	58.86 (9.07)	56.98 (7.33)

## Discussion

Recent research highlights the need to improve and evaluate patient empowerment in persons with CCs [4]. However, the availability of instruments with a strong theoretical background and/or acceptable psychometric properties is limited [6]. Therefore, the present study evaluated a slightly modified version of a previously validated patient empowerment in a group of adults with CHD. Results indicate that GES proved reliable, meaning the scale’s items altogether are consistent. Additionally, no floor or ceiling effects were identified, which is indirectly related to the scale’s sensitivity to measure change [22]. Whereas these results are in favor of the GES, a psychometric assessment of the scale’s validity revealed that the scale appears valid in two of the three included countries.

Results from the CFA of the entire sample indicate the five-factor structure fits the data well. However, due to the lack of measurement invariance, CFAs were performed separately for patients from each country. Analyses revealed that an acceptable model fit was only achieved for the samples from Belgium and Norway. For the South Korean data, the CFI and the RMSEA were above the acceptable ranges although factor loadings from the “shared decision-making” dimension were low. This is an indication that the relations among the latent constructs of GES are not in line with the data, i.e., the data do not support the model [24]. Even though it is common in CFA to conduct an evaluation of modification indexes to improve model fit, changes should be theoretically justified [25]. In this case, we could identify no strong theoretical reasons to allow for model changes that could potentially improve the model fit.

The poorly fitted model to the South Korean data might be the reason why invariance was not achieved when comparing the three countries. A lack of invariance suggests that patient empowerment may have a different meaning in different countries. Therefore, making mean comparisons of this construct across countries using the GES should be made with caution. Nonetheless, while cross-country comparisons cannot be made, it is possible to compare how the GES relates to other constructs (i.e., variables) *within* countries. For example, one could undertake a study to compare how the GES relates to patient functioning in Belgium, vs. how the GES relates to patient functioning in South Korea. Another potential research aim would be to determine whether changes in GES relate to psychological distress and whether these changes have the same magnitude in Belgium, Norway and South Korea.

The absence of invariance across the three countries can be an indicator of patient empowerment being interpreted differently across countries. Perhaps individuals in South Korea have different interpretations and values of patient empowerment compared to patients in Belgium and Norway, two European countries that are likely more culturally similar. It has been suggested that patient empowerment is determined by its context and that individuals within a particular environment, organization or country may have a different perception of empowerment [26]. Hence, a measure that fits all persons (or contexts), might be hard to develop. Findings from the present study partially support this notion. It is plausible to consider that different countries (and cultures) have a different understanding of patient empowerment domains, values, and skills.

Evidence on how patient empowerment may differ between countries is limited. However, authors of a systematic review concluded that although patients do share many perspectives of this construct, there might be variations on how certain aspects of patient empowerment are understood and research has yet to address structural elements of patient empowerment [27].

Consideration of structural elements is relevant for the current study because the “shared decision-making” dimension had the lowest factor loadings for the South Korean sample, as well as poor reliability. Shared decision-making involves healthcare professionals and patients working together on a care plan [28]. It entails patients who are willing to participate as well as clinicians who want to collaborate with the patient. Hence, it is greatly influenced by the healthcare structure. Within Asian cultures, for example, it is more commonly believed that patients prefer to take a less independent role and that a family-centered (rather than individual-centered) approach towards decision-making is preferred [29–31]. It has been suggested that shared decision-making reflects values associated with western cultures and that this might be different in other cultures [28, 31, 32]. The value placed on shared decision-making and the structure of the healthcare system might therefore potentially impact the value of patient empowerment as perceived by study participants. Therefore, the comparison of this construct between different cultures and regions of the world should be made with caution.

Although the GES appears to be a valid and reliable instrument for the Norwegian and Belgian sample, it is worth noting that future research should evaluate whether the low psychometric properties found in the South Korean sample are indeed applicable to other Asian or non-European countries. Comparatives studies evaluating this construct (and similar constructs) between Western and Asian countries are needed to comprehend this phenomenon, the types of research questions that can be addressed and the conclusions that can be drawn when evaluating outcomes such as patient empowerment. Such studies could be undertaken once the data collection from APPROACH-IS II is finalized.

### Methodological considerations

There are several strengths of the study that merit mentioning. First, GES development was based on a previously validated questionnaire, which in turn has a strong theoretical foundation and followed a rigorous development process. Therefore, it is plausible to conclude that GES also has a strong theoretical foundation. Second, data were collected as part of an international study, which allows for cross-country comparisons. Third, the data were collected before the COVID-19 pandemic, therefore there was no concern about the potential for survey responses to be impacted by the pandemic [33].

There are, however, also some methodological limitations associated with this study. First, some aspects associated with validity and reliability were not assessed. For instance, it was not possible to directly evaluate responsiveness with cross-sectional data. While floor and ceiling effects are an indirect way to measure responsiveness, this is better achieved through longitudinal studies. Future longitudinal studies should evaluate this psychometric property. Second, the study only includes individuals with CHD, who were in current follow-up and who had the abilities to answer the questionnaires. Therefore, the generalizability of the results might be limited.

## Conclusion

This study provides evidence on the GES’s validity, reliability, and responsiveness in adults with CHD. GES seems to be valid and reliable for the sample of Belgium and Norway. However, this is not the case for South Korea, because shared decision-making seems to have a different meaning in Asian cultures. Hence, cross-cultural comparisons using GES should be made cautiously. It is possible that in countries who are more culturally different from Norway or Belgium, the scale might not perform as well.

## Data Availability

The data analyzed in the current study are available from the corresponding author on reasonable request.

## Abbreviations

CCs: chronic conditions.
CFA: confirmatory factor analyses.
CFI: comparative fit index.
CHD: congenital heart disease.
GES: Gothenburg Empowerment Scale.
GYPES: Gothenburg Young Persons Empowerment Scale.
MGCFA: multigroup confirmatory factor analyses.
RMSEA: root mean square error of approximation.
SRMR: standardized root mean square residual.
